# The Public and Venereal Diseases

**Published:** 1910-08

**Authors:** R. H. Stanley

**Affiliations:** Albany, Ga.


### THE PUBLIC AND VENEREAL DISEASES.

BY R. H. STANLEY, M. D. ALBANY, GA.

In the presentation of this paper, I expect to develop no new theories for your consideration. The facts herein stated are perhaps familiar. I have accomplished my purpose if I can place familiar facts before you in such shape that you will become aroused to a full realization of their importance.

We Americans are faddists. There is within us an innate force which compels us to be ever exercised over some economical problem of real or imaginary importance. It seems the natural outlet for our superabundant energy and probably saves us from drifting into innocuous dissuetude. The medical profession is not exempt. If we haven't the child labor problem we have appendicitis, and if we haven't appendicitis we have tuberculosis. If these weary us, we may turn to the more strictly sectional pellagra or its rival, the hook worm. What the next will be, the Lord only knows. Nor would I utter a syllable that would seem to cast a doubt upon the value of such upheavals or hinder the progress of social reforms; but while we are training our big guns upon these relatively newer enemies of the human race, we are weakening our defenses against those we have had with us from time immemorial and whose toll of human life is equally as great.

Ranking third in the list of diseases as the cause of death, as it does, and on account of its close relationship to every other branch of the medical science, venereal disease in its varied manifestations is a problem with which it is vicious to temporize and which it is criminal to neglect.

Are we doing our full duty toward the public relative to the venereal troubles? Is it not a fact that our tolerance, indifference and avoidance of a disagreeable subject has engendered in the minds of the laity the idea that a gonorrhœa is hardly worse than a coryza and that syphilis is only more annoying from the standpoint of long treatment?

Let us review, if you please, what the leading specialists in the profession have to say concerning the spread and destructiveness of gonorrhœa and syphilis. Forscheimer affirms that fifty-one per cent. of the male population have gonorrhœa, while Morrow goes further and places the percentage at sixty. In 1907 the committee of seven, appointed by the American Medical Association to investigate the prevalence of the disease, reported that there were apparently 162,372 persons suffering from venereal troubles in New York City. Of 15,969 cases of gonorrhœa reported there were 1941 in women—and we know that after it passes the vagina it is hard to detect—and 488 cases in children. About one-third of the cases occurred in married women to whom the infection had been conveyed by their husbands.

As far back as 1876, in a paper read before the American Gynecological Society, Noeggarath claimed that ninety per cent. of sterile women are married to husbands who have suffered from gonorrhœa previous to or during married life and his assertion has not been successfully contradicted. Incidentally he claimed that gonorrhœa persists during life and that view is held by many noted scientists.

Valentine and Townsend claim that whenever the posterior urethra becomes infected acute gonorrhœal prostatitis invariably follows. The gonococcus finds in the prostate a fortified castle where it may abide indefinitely and from his safe retreat make excursions from time to time into the urethra to light up anew the canal. Such infections explain the frequent return of the disease in patients apparently cured.

Keys reports two patients who, without apparent re-infection, continued to show gonococci in the prostatic secretion for two years from the beginning of the disease, and in another patient the germ was present for three years. I was recently consulted by a young man who stated that with short intermissions he had suffered from a gonorrhœal discharge continuously for nearly two years.

The malady is of even more sinister import to woman. Eliminate it from the category of her ailments and the gynecologist would have to seek other fields of endeavor. The committee of seven, once before quoted, reported that eighty per cent. of the deaths from female pelvic diseases are due to gonococcic infection. Once in the uterus and tubes we can never be sure of a cure and so long as the uterus and appendages are thus diseased is it possible to convey the infection.

Neisser and Bumm hold that gonorrhœa is responsible for thirty per cent. of all cases of sterility in women, while Morrow raises the percentage to fifty.

Since the discovery of the gonococcus by Neisser in 1879, the study of the disease has been placed upon a scientific basis and the microscope has revealed the fact that it is responsible for many symptoms that were formerly accredited to other causes. The seriousness of its systemic disturbances are in many ways similar to syphilis.

The gonococcus has been found in the submucous tissues by Mandl; in the squamous epithelium by Bumm; in the thromboid blood vessels of the bladder, in the serosa of the peritoneum and in the substance of the ovary by Wortheim; in the connective tissues of the uterine tubes by Kraus; in the rectal mucous membrane by Fritsch; in the uterine muscle by Madlener. Kronig found gonococci in the lochia of over ten per cent. of 296 lying-in women who had childbed fever. It has been found in the pus of inflamed joints by Stern and in the pus of an inflamed tendon sheath by Kronig. Lenhartz has isolated it from the thrombus of the pulmonary valve in a case of ulcerative endacarditis and it has been found in the blood current by Leyden and others.

I have dwelt at length upon gonorrhœa for the reason that the laity and, unfortunately, many physicians consider it a local disease of minor importance.

In the study of syphilis through the medium of modern scientific research, we find the ravages of the disease more general and the outlook more discouraging.

The uncertainty that has heretofore existed has been swept away by the Wassermann reaction and the discovery of the spirochata pallida, and where we have been giving different names to the various manifestations we are now able to classify them all under the symptom complex of syphilis.

In his Shorstein lecture, Osler estimated the mortality from syphilis at about one to eighty of all deaths, and he does not take into account the vast number of stillbirths. He places the number of deaths as a direct result of the disease at one-seventh, while anurysm and the enormous group of affections of the nervous system represent later but none the less definite results of the poison.

If the destructiveness of the disease was confined to the individual, the situation would be sufficiently serious, but unfortunately it has an incomparably more fatal influence upon society as a whole.

According to Fournier's well-known statistics, when the father alone is actively syphilitic, sixty-seven per cent. of the children are born syphilitic and twenty-eight per cent. die. If the mother alone is syphilitic, eighty-four per cent. of the children are born so and sixty-eight per cent. die. If both parents are syphilitic, as is usually the case, ninety-two per cent. are born infected.

The Census Bureau stated that in 1908 inherited syphilis claimed more than one-eighth million babies under one year of age and 200,000 children under five years of age among about one-half of the total population of the United States.

One infection does not render the patient immune and syphilographers practically all concur in the statement that hereditary syphilis gradually acquires a susceptability and that the immunity is extinguished about puberty.

There is no organ in the human system that may not be attacked by syphilis. Its fatal course may terminate in a few months or it may stretch out into years of chronic bone and visceral degeneration. It is apparently the most common cause of aneurysm. It is a frequent cause of early arterio-sclorosis. It is practically the sole cause of tabes and paresis and figures largely in the etiology of apoplexy, paraplegia and insanity.

It may be transmitted by practically all of the secretions and excretions of the body.

The remedy lies in education and it devolves upon us as self-imposed guardians of the public health to act as tutors.

Pity it is that the framers of our organic law did not profit by Biblical teaching and include in our constitution the fifteenth chapter of Leviticus. On the contrary, however, our benevolent government sends an army of skilled men to eradicate any other contagion but this. It is a weak spot in our governmental system that we can do much to strengthen. We may not expect to accomplish great things with the present generation, but the good seed can be sown and the harvest will be reaped by the generations yet unborn. We sacrifice ourselves upon the altar of public good by preaching and teaching preventive medicine and leave our pupils in ignorance on a subject that presents itself every day in our routine work.

We should use our influence to see that such teachers are secured for our public schools who have the moral courage to defy a sickly public sentimentality and dare to instruct their charges on the origin of life and the physiology and hygiene of self. We should teach our young men patients that sexual intercourse is not essential to health and arouse the public to the dread dangers that lurk in prostitution, the darkest blot upon our civilization.

If venereal diseases were put in the same category with other acute infections; if a stricter surveillance was placed upon the prostitutes, and if the defectives in our prisons and houses of detention were treated by the Indiana method of sterilization, there would be a rapid diminution of the peril that now threatens the nation.

When Twashtri found that he had exhausted his solid elements in creating man, after profound meditation he took the rotundity of the moon and the curves of the creepers, and the clinging of the tendrils and the trembling of grass, and the slenderness of the reed and the bloom of flowers, and the lightness of leaves and the tapering of the elephant's trunk, and the glances of the deer and the clustering of rows of bees, and the joyous gayety of the sunbeams and the weeping of clouds, and the fickleness of the winds and the timidity of the hare, and the vanity of the peacock and the softness of the parrot's bosom, and the hardness of adamant and the sweetness of honey, and the cruelty of the tiger and the warm glow of fire, and the coldness of the snow and the chattering of jays, and the cooing of the kokila and the hypocrisy of the crane, and the fidelity of the chakrawaka, and compounding all of these together he created woman and called her his masterpiece. This woman, radiant in the glow of health and beauty, as innocent as the dove and as pure in thought, sips at the fountain of love whose stream has been poisoned through ignorance in a former lust and gives up her young life a sacrifice, and thus it is that the missed opportunity of the physician to do his full duty in uttering kindly words of warning to adolescent youth, leaves through the world a train of misery and death.

